# Expression of biologically active bovine interleukin 7 and evaluating the activity *in vitro*

**DOI:** 10.14202/vetworld.2016.160-165

**Published:** 2016-02-13

**Authors:** J. Lijo, N. Vijay, H. J. Dechamma, G. R. Reddy

**Affiliations:** FMD Research Laboratory, Indian Veterinary Research Institute, Hebbal, Bengaluru, Karnataka, India

**Keywords:** B-cell lymphoma 2, nuclear factor for activated T-cells c1, recombinant bovine interleukin 7, signal transducer and activator 3

## Abstract

**Aim:**

Interleukin 7 (IL-7) is a ϒc family cytokine involved in the homeostatic proliferation and maintenance of immune cells. In the present study, we report the expression of bovine IL-7 (bIL-7) in *Escherichia coli* and evaluated for its biological activity.

**Materials and Methods:**

The sequence coding for bIL-7 (mature protein) was amplified from primary bovine kidney cell culture and cloned into pET28-a vector and expressed in *E. coli* (BL 21 DE3). The expressed protein was purified by nickel-nitrilotriacetatechromatography, and the reactivity of the protein was confirmed by western blotting using monoclonal antibodies raised against human IL-7. The biological activity of expressed bIL-7 was evaluated by analyzing its effect on the expression of anuclear factor for activated T-cells c1 (NFATc1), B-cell lymphoma 2 (Bcl2), suppressor of cytokine signaling 3 (SOCS3) molecules in bovine peripheral blood mononuclear cells (PBMCs) by quantitative polymerase chain reaction. Ability of the expressed protein was also analyzed by its effect on phosphorylating signal transducer and activator 3 (STAT3) molecule by immunostaining in human embryonic kidney cells 293 (HEK293) cells.

**Results:**

The bIL-7 was able to induce the expression of Bcl2 and NFATc1expression in bovine PBMCs by 7 and 5-folds, respectively, whereas a 2-fold decrease was observed in the case of SOCS3 expression. Immunostaining studies in HEK293 cells using antihuman phospho-STAT3 showed activation and nuclear translocation of STAT3 molecule on bIL-7 treatment.

**Conclusion:**

bIL-7 gene was successfully amplified, cloned, and expressed in a prokaryotic expression system. The biological activity study showed that the *E. coli* expressed bIL-7 protein is biologically active. Considering the role of IL-7 in T-cell homeostasis and memory cell generation, this molecule can be used for enhancing the vaccine response and that has to be proved by further experiments.

## Introduction

Interleukin 7 (IL-7) is a vital cytokine for the homeostasis and development of both T- and B-lymphocytes. It is produced by intestinal epithelial cells, keratinocytes, hepatic tissues, peripheral blood dendritic cells, endothelial cells, smooth muscles, and fibroblast cells in human, however in mouse, thymic stromal cells, fetal thymus cells, bone marrow, spleen, kidney, keratinocytes, and fetal intestine are identified as the sites of IL-7 production [1]. IL-7 is a member of an IL-2 group of interleukins, and it effects through a receptor complex consisting of IL-7 receptor alpha chain (IL-7Rα) and ϒc chains, where ϒc chain is common for the IL-2 group of interleukins [2,3]. Binding of IL-7 to IL-7Rα-ϒc complex activates Janus activated kinase-signal transducer and activator (JAK-STAT), PI3-Akt pathways, and Src family of protein kinases [1,4].

IL-7 activation promotes survival and proliferation of lymphocytes [5]. The survival effect of IL-7 is mainly mediated by activation of B-cell lymphoma 2 (Bcl2) protein through JAK-STAT pathway [6]. It also activates other Bcl-2 family proteins, Bcl-X_L,_ and MCI-1, which are also anti-apoptotic in nature [2,7]. Suppression of pro-apoptotic proteins is another way by which IL-7 improves the survival of lymphocytes [1]. Apart from the anti-apoptotic activities, IL-7 regulate the metabolism and pH in T-cells [8,9]. The most important function of IL-7 is the production and homeostatic maintenance of memory T-cells [10]. Very recent reports say that IL-7 increases the longevity of memory cell by enhancing triacylglycerol synthesis by promoting aquaporin 9 [11].

The crystal structure of IL-7 receptor and their binding properties with IL-7 were well-studied in the case of human IL-7 [12].

Since there is no report on the characterization of bovine IL-7 (bIL-7), the present study reports the cloning of full-length sequence of bIL-7 and expression of the bIL-7 mature (bIL-7 mat) protein in prokaryotic expression system and evaluation of its biological activity *in vitro*.

## Materials and Methods

### Ethical approval

All the procedures were carried out with prior ethical permission of Institute Bio-Safety Committee (IBSC) and Institute Animal Ethics Committee (IAEC), Indian Veterinary Research Institute, Hebbal, Bangalore. (Ref.No. F.8-56-Vol.II/RCSS/2014-15/dtd 8-06-2015).

### Amplification and cloning of bIL-7

Bovine kidney cells were collected in normal saline from the local slaughter house, and bovine primary kidney cell culture was prepared. Total RNA was isolated from the primary bovine kidney cell culture using TRIZOL reagent (Invitrogen, Carlsbad, USA).cDNA was prepared by reverse transcriptase reaction using oligo dT primers. The cDNA was used to amplify the IL-7 full-length sequence using IL-7L (5’-ggcgggtaccatgttccatgtttcttttagg-3’) and IL-7R (5’-ggcggcggccgctcagtgttctttaatgccc-3’) primers which were designed using IL-7 sequence available in the database (Gene Bank Accession No: BC142466.1). The purified polymerase chain reaction (PCR) product was ligated into EcoRV digested pcDNA (+3.1) vector. The cloned insert was subjected for sequencing by an automated sequencer, and the sequence was confirmed by comparing with known related sequences present in the database by NCBI blast tool.

### Expression of mature IL-7 in *Escherichia coli*

bIL-7 mat sequence was amplified from the full-length IL-7-pcDNA plasmid using IL-7Lmat (5’-gcggatccgattgtgatattagaggtaaagac-3’) and IL-7R (5’-ggcggcggccgctcagtgttctttaatgccc-3’) primers. The PCR product was cloned into pET28a vector at BamHI and NotI sites to generate pET28-bIL-7 mat and transformed into DH5α cells. The recombinant plasmid pET28-bIL-7mat confirmed by restriction digestion analysis and expressed in BL21c DE3 strain of *E.coli*. Overnight grown cultures of four randomly selected recombinant colonies were inoculated to 10 ml Luria-Bertani broth medium and grown to optical density_600_= 0.6-0.8 at 37°C. Protein expression was induced with 1 mM isopropyl thiogalactoside (IPTG) for 5 h at 30°C. The pellet was lysed and analyzed by 10% sodium dodecyl sulfate-polyacrylamide gel electrophoresis (SDS-PAGE) gel for protein expression under denaturing conditions. After confirming the protein expression, the fusion protein was purified by nickel-nitrilotriacetate (Ni-NTA) chromatography (Nucleopore, USA) using denaturing conditions for eluting the protein by elution buffer containing 250 mM imidazole. Protein was partially refolded by reducing the molarity of urea in the washing buffer and different fractions of elute were analyzed by SDS-PAGE. The purified samples were ran on 10% SDS-PAGE and transferred to nitocellulose membrane for western blotting using human Il-7 specific monoclonal antibodies (Biolegend, USA).

### Biological activity of expressed IL-7

The biological activity of purified mature IL-7 was studied by its effects on expression of Bcl2, nuclear factor for activated T-cells c1 (NFATc1), and suppressor of cytokine signaling 3 (SOCS3) molecules. Peripheral blood mononuclear cells (PBMCs) were isolated from cattle blood using histopaque 1077 (Sigma, USA). Approximately 2 × 10^6^ cells were dispersed in each well in a six-well plate. About 100 ng of the recombinant protein was used per well for the biological activity assay. PBMCs were incubated with mature IL-7 for 3 h, and total mRNA was collected by TRIzol reagent (Life technologies, USA). cDNA was synthesized using oligodT primers. Real time primers were designed for Bcl2 (forward 5’-agcgtcaaccgggagatgt-3’, reverse 5’-tagggccatacagctccac-3’), NFATc1 (forward 5’-caggtgagtccgacgtcaag-3’, reverse 5’-gtcagctctcgggtccca-3’), and SOCS3 (forward 5’-catctctgtcggaagaccgt-3’, reverse 5’-taaagcggggcatcgtactg-3’) molecules using Primer blast software of NCBI. ABI prism 7300 (Applied Biosystems) was used to carry out qPCR with conditions consisting of an initial step at 50°C for 2 min and 95°C for 2 min followed by 40 cycles at 95°C for 15 s and at 60°C for 1 min. The qPCR results were analyzed by relative quantification method [13], where glyceraldehydes 3 phosphate dehydrogenase (GAPDH) was used as an internal control gene.

Immunostaining analysis of STAT3 molecule activation was carried out in a six-well culture plate. Approximately 1 × 10^5^ human embryonic kidney cells 293 (HEK293) cell per well were seeded in six-well plate and allowed to grow for 24-36 h. Cells were incubated with 10 μg/ml of purified *E.coli* expressed IL-7 mature protein for 30 min. After incubation cells were washed twice with 1 × phosphate buffered saline (PBS) and fixed with 3.7% formaldehyde in 1 × PBS (NaCl- 137 mM, KCl- 2.7 mM, Na_2_HPO_4_- 10 mM, and KH_2_PO_4_- 2 mM) for 15 min. The plate was again washed twice with 1 × PBS and treated with 0.5% triton x-100 (1 × PBS) for 15-20 min to permeabilize the cell and nuclear membrane. Cells were washed twice with 1 × tris-buffered saline (TBS)-Tween (Tris-Cl pH 7.4-10 mM, NaCl- 0.9%, Tween 20-0.02%) and blocked with 3%BSA (in TBS-Tween) for 1h. Washing was repeated thrice with 1 × TBS-Tween and incubated with the anti human phospho-STAT3 antibody (1:1000 in TBS-Tween) for 1 h at 37°C. After washing secondary antibody conjugated with fluorescein isothiocyanate (1:2000 in 1 × TBS-Tween) was added to the plates and incubated for 1h at 37°C. The plates were washed with 1 ×TBS-Tween and observed under fluorescence microscope.

## Results

### Biochemical characterization of recombinant bIL-7 mat

**Figure-1:**
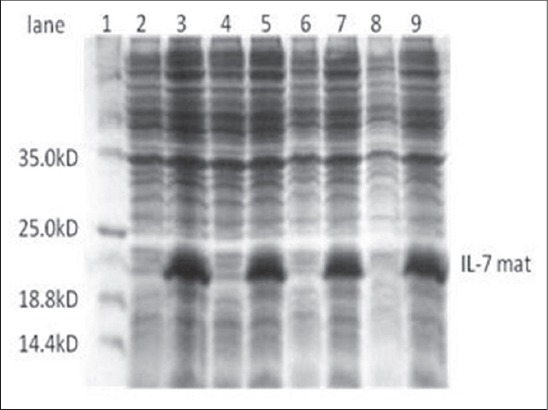
Polyacrylamide gel electrophoresis analysis of recombinant BL21c *Escherichia coli* colonies for bovine interleukin 7 mature protein expression. Randomly selected overnight grown cultures of recombinant colonies were inoculated to 10 ml Luria-Bertani broth medium and grown to optical density_600_= 0.6-0.8 at 37°C. The culture was induced with 1 m Misopropyl thiogalactosidasefor 5 h at 30°C. The pellet was lysed and analyzed by 10% sodium dodecyl sulfate-polyacrylamide gel electrophoresis gel for protein expression under denaturing conditions. Lane 1: Molecular weight protein markers, Lane 2, 4, 6, 8: Cell lysate of uninduced recombinant PET28-boIL-7mat/BL21c *E. coli* colonies, Lane 3, 5, 7, 9: Cell lysate of recombinant PET28-boIL-7mat/BL21c *E. coli* colonies after induction.

**Figure-2:**
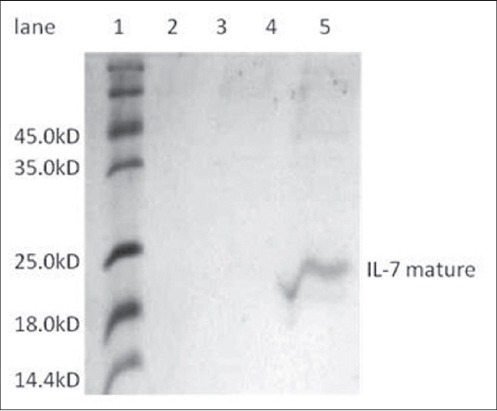
Polyacrylamide gel electrophoresis analysis of purified bovine interleukin 7 mature (bIL-7 mat) protein purified by nickel-nitrilotriacetate (Ni-NTA) chromatography. pET28a expressed bIL-7 mat protein was purified by Ni-NTA chromatography. After loading the protein, column was washed with reducing concentrations of urea in wash buffer and eluted with 250 mM imidazole. Different fractions of elute loaded for checking the presence of protein. Lane 1: Molecular weight protein markers, Lane 2, 3, 4, 5: Differentelutes of Ni-NTA chromatography.

**Figure-3:**
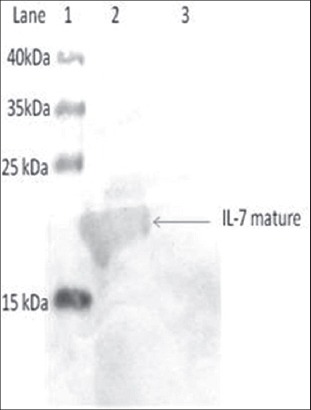
Western blotting analysis of recombinant bovine interleukin 7 mature (bIL-7 mat) protein. Immunoblotting of recombinant bIL-7 matprotein using antihuman IL-7 antibodies. Lane 1: Molecular weight protein markers, Lane 2: Purified IL-7 mature protein, and Lane 3: BL21 cell control.

In this study, the recombinant plasmid was transformed into BL21c DE3 strain of *E.coli*, and protein expression was induced by 1mM IPTG. On SDS-PAGE analysis, an extra band of 20 kDa was observed in induced cultures when compared with uninduced cultures (Figure-1). Since the protein was tagged with histidine, the mature IL-7 protein was purified by Ni-NTA affinity column. Upon analyzing the different fractions of elute by SDS-PAGE (Figure-2), a single band (Lane 5) corresponding to IL-7 mature protein was observed. The elute was re-natured by dialysis and the purified IL-7 mature protein further characterized by immune blottingusing human specific IL-7 monoclonal antibody (Biolegend, USA) (Figure-3).A thick band around 20 kDa and a light band around 40 kDa were seen reacting with IL-7 specific sera suggesting the reactivity of the protein (Figure-3).

### Biological activity of *E.coli* expressed bIL-7

**Figure-4:**
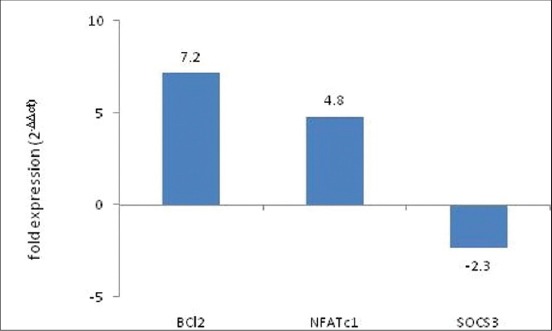
Biological activity study of bovine interleukin 7 mature (bIL-7 mat) protein in bovine peripheral blood mononuclear cells by quantitative polymerase chain reaction. Approximately 2 × 10^6^ cells were incubated with 100 ng of bIL-7 matprotein for 3 h. Copies of different mRNA molecules were quantified by real time polymerase chain reaction. B-cell lymphoma 2 and nuclear factor for activated T-cells c1 molecules show 7.2- and 4.8-fold increase while suppressor of cytokine signaling 3 molecules 2.3-fold decrease in the treatment group when compared with control group.

Biological activity of *E.coli* expressed bIL-7 was evaluated by assessing itseffect on the expression of Bcl2, NFATc1, and SOCS3 molecules. In this study, the effect of bacterial expressed IL-7 on these molecules was analyzed by qPCR by keeping GAPDH gene as an internal control. The results showed 7.2-fold increase in the expression of Bcl2, 4.8-fold increase in the NFATc expression, and 2.3-fold decrease in the expression of SOCS3 compared to the control in bovine PBMC culture (Figure-4).

**Figure-5:**
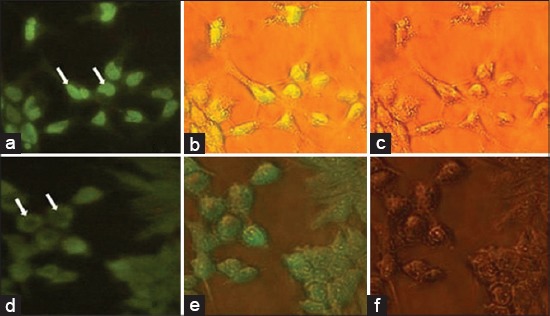
Immunostaining analysis of signal transducer and activator 3 (STAT3) phosphorylation by bacterial expressed bovine interleukin 7 (IL-7) matureprotein (400 × magnifications). 10^5^ cells per well were seeded in six-well plates and incubated with 10 μg/ml recombinant IL-7 mature protein. Cells were fixed with formaldehyde and permeabilized with Triton-100. Cells were incubated with anti-phospho-STAT3 (Tyr705) antibodies and conjugated with secondary antibody tagged with fluorescein isothiocyanate (FITC) and visualized under fluorescent microscope. (a) cells under ultra-violet [UV] light shows more fluorescence in the nucleus than the cytoplasm, (b) cells were visualized under both UV and plain light, (c) cells illuminated with plain light showing no fluorescence, (d) Control cells show less fluorescence compared nueclues when illuminated under UV light, (e) same cells visualized under UV and plain light, (f) cells visualized under plain light shows no fluorescence. Control cells not showing any fluorescence in the nucleus and only cytoplasm is stained.Cells incubated with recombinant IL-7 mature protein show fluorescence in the nucleus showing localization of STAT3 molecules in the nucleus.

The effect of *E.coli* expressed IL-7 on phosphorylating STAT3 molecule is analyzed by immunostaining. HEK293 cells were incubated with 10 μg/ml of recombinant protein for 30 min. Later cells were processed for immunostaining according to the above-described procedure. Cells incubated with recombinant bIL-7 showed prominent fluorescence in the nucleus (Figure-5a, 5b and c), whereas the control cells show fluorescence only in the cytoplasm, not in the nucleus (Figure-5d, 5e and f).

## Discussion

IL-7 is believed to be the most important cytokine involved in the homeostatic proliferation of T- and B-cells. IL-7 signals through a receptor complex of IL-7Rα and γ chains [3]. IL-7 is a 20 kDa protein containing 176 amino acids in length which is further processed into active mature protein with 151 amino acids. In the present study, the sequence coding for bIL-7 mat was amplified, expressed in prokaryotic expression system and the specificity was confirmed by western blotting using human IL-7 specific monoclonal antibodies. In the blot, we could observe a thick band around 20 kDa size, including 3 kDa tag from pET28a vector, which is of expected molecular weight of the mature IL-7 protein.

Homeostatic proliferation and survival of immune cells are mainly attributed to the function of IL-7. The survival effect of IL-7 is mainly elicited by up-regulating anti-apoptotic proteins such asBcl2-family proteins by the JAK3/STAT5 pathway. Expression of Bcl2 decreases in effector cells and increases to the maximum in memory cells, when compared to naïve CD8^+^T-cells [14]. The expression of IL-7R and Bcl2 may play an important role in cells which transform from effector to memory cells. NFAT family of protein consists of four transcriptional factors, among which NFATc1 (NFAT2) plays a major role in T-cell activation. Upon activation, NFATc1 translocates to the nucleus and binds to promoters and enhancers of many interleukin genes resulting in the activation of T-lymphocytes. In general, NFATc1 regulated by calcium-calmodulin-calcineurin pathway leads to dephosphorylation of NFAT and nuclear translocation [15,16]. Recently, an alternative pathway of activation of NFATc1 by IL-7 has been elucidated [17]. A parallel IL-7-JAK3-NFATc1 signaling occurs to the conventional IL-7-JAK3-STAT5 pathway. Phosphorylation of Tyr371 residue occurs in IL-7-mediated activation of NFATc1 instead of dephosphorylation in conventional calcineurin-mediated activation of NFATc1. In this study, we observed an up-regulation of NFATc1 at the transcription level on IL-7 treatment.The current study showed a 7.2- and 4.8-fold increase in Bcl2 and NFATc1 expression, respectively. The IL-7-jak3-stat5 pathway is the way in which Bcl2 is activated [6],and the same result is repeated in the current experiment. We suggest that phosphorylation of IL-7 at Tyr371 may have a positive feedback on the NFATc1 expression and that has to be confirmed with further experiments.

SOCS molecules are the physiological suppressors of cytokine signaling. They function as internal regulators of cytokine signaling, and many of the SOCS molecules are activated as a response to cytokine signaling. Because of their cytokine regulatory function, SOCS molecules are exploited by many infectious agents to escape from host immune system. It has been shown that IL-7 therapy augments IL-6 production by inhibiting SOCS3 activation by regulating FoxO transcriptional activity[18]. Our result supports these observations as bIL-7 causes a 2.3-fold reduction in the expression of SOCS3 molecule in PBMC cells.

The effect of *E.coli* expressed IL-7 on phosphorylating STAT3 molecule is analyzed by immunostaining. In the present experiment, HEK293 cells incubated with IL-7 showed fluorescence in the nucleus indicating the nuclear translocation of the phosphorylated STAT3 molecules,whereasthe nucleus of the control cells did not show any fluorescence and remained restricted to the cytoplasm. On activation, STAT molecules would be translocated into the nucleus and elicit effects at the transcription level. Phosphorylation of STAT molecule is one of the modes of action of IL-7. STAT5 is documented as the major candidate for the JAK/STAT signaling pathway by IL-7. In the development of B-cells, especially pre-B-cells depend on IL-7-mediated STAT3 signaling. Studies revealed that STAT3 deficient pre-B-cells are highly susceptible for apoptosis [19]. IL-7 increases the absolute number of γδ^27-^thymocytesin adult mice and promote the capacity of γδ^27-^ to produce IL-17, which help in the polarization of T-cells. This selectivity of IL-7 on γδ^27-^thymocytes to produce IL-7 mainly owes for its capacity to activate STAT3 in such cells [20]. Like STAT3, STAT5 also activated (phosphorylation of STAT5) by IL-7. STAT5 along with properly balanced PI3/AKT enhances the memory cell survival [21].

Imperative role by IL-7 in T-cell memory prompted the molecule to be tested for its adjuvanted effect in its DNA and protein forms. It showed increased T-cell response against hepatitis C virus infection [22] and avian influenza [23]. IL-7tv, a modified IL-7 protein, which has a lower binding capacity than IL-7 with IL-7R successfully promoted CD4 and CD8 populations and reduced the IL-7 receptor down-regulation due to supra physiologic doses of IL-7 [24]. It is suggested that IL-7 doses should be intermittent to reduce the IFN-γ-mediated ­apoptosis [25]. IL-7 seems to be a promising molecule to be studied and tested further for increasing the effectiveness of vaccines, especially against viral infections.

## Conclusion

The present study shows that *E.coli* expressed bIL-7 matare biologically active. Uponstimulation with the recombinant bIL-7, there was an increase in the mRNA levels of NFATc1 and Bcl2 and a decrease in the expression of the SOCS3 molecule in PBMCs. Use of IL-7 protein and IL-7 encoding DNA constructs are reported to be useful against cancer and many viral infections. Biologically active bIL-7 expressed in *E.coli* will be a good candidate to evaluate further it as a therapeutic agent as well as an adjuvant to the vaccine in bovines.

## Author's Contributions

JL- The research work mentioned in this article is a part of the Ph.D research work of the first author and first author carried out all the work mentioned in this article. NV - The immunostaining of HEK cells to analyze the biological activity of the bIL-7 protein. JHD - The real time PCR assay described in the present study. GRR- The entire work mentioned in this article was carried out under the guidance of the fourth author.
